# A novel non-sulphamoylated 2-methoxyestradiol derivative causes detachment of breast cancer cells by rapid disassembly of focal adhesions

**DOI:** 10.1186/s12935-018-0688-7

**Published:** 2018-11-19

**Authors:** Mandie Botes, Tamarin Jurgens, Zohreh Riahi, Michelle Visagie, Rustelle Janse van Vuuren, Anna Margaretha Joubert, Iman van den Bout

**Affiliations:** 1 Department of Physiology, University of Pretoria, Pretoria, 0084 South Africa; 2 Centre for Neuroendocrinology, University of Pretoria, Pretoria, 0084 South Africa

**Keywords:** Breast cancer, 2-Methoxyestradiol, Cell detachment, Focal adhesion

## Abstract

**Background:**

2-Methoxyestradiol (2ME2) is an estradiol metabolite with well documented antiproliferative properties in many cancer cell lines. However, it is rapidly metabolised in vivo which limits its clinical application. Therefore, more stable derivatives with potentially improved clinical features have been designed by our group. Here we describe an estrone-like derivative of 2ME2, namely EE-15-one, that unlike other derivatives which induce cell cycle arrest, induces a rapid loss of cell–substrate adhesion through the inactivation and disassembly of focal adhesions.

**Methods:**

To assess the effect of 2-ethyl-estra-1,3,5 (10),15-tetraen-3-ol-17-one (EE-15-one) on breast cancer cell lines, cell survival was quantified. The effect of EE-15-one on cell attachment was assessed by measuring cell adhesion and cell rounding via light microscopy. Effects on focal adhesion dynamics and actin cytoskeleton organisation were visualised by immunofluorescence while focal adhesion signalling was assessed by western blot. Cell death was quantified using a lactate dehydrogenase activity (LDH) assay. To investigate specificity towards cell–substrate over cell–cell contact inhibition, EE-15-one effects on 3D cell cultures were assessed.

**Results:**

Cell survival assays show an almost complete loss of cells within 24 h of EE-15-one exposure in contrast to published sulphamoylated 2ME2 derivatives. Cell loss is linked to rapid detachment and adhesion inhibition. Focal adhesion size and number are rapidly diminished while actin fibres became severed and disappeared within 2 h post exposure. These changes were not due to cell necrosis as LDH activity only slightly increased after 24 h. Cells grown in cell–cell adhesion dependent spheroids did not respond to EE-15-one exposure suggesting that EE-15-one specifically inhibits cell–substrate adhesions but not cell–cell adhesions and does not directly impact the actin cytoskeleton.

**Conclusion:**

We show that a novel 2ME2 derivative, EE-15-one, induces rapid loss of focal adhesion function leading to cell–substrate detachment through interference with integrin-based cell–substrate adhesions, but not cadherin dependent cell–cell adhesions. Therefore, EE-15-one is the first 2ME2 derivative that has an alternative mode of action to the antimitotic activity of 2ME2. As such EE-15-one shows potential as a lead compound for further development as an inhibitor of cell–substrate adhesion which is essential for metastatic dissemination.

**Electronic supplementary material:**

The online version of this article (10.1186/s12935-018-0688-7) contains supplementary material, which is available to authorized users.

## Background

2-Methoxyestradiol (2ME2) is an oestrogen metabolite that exhibits antiangiogenic and antiproliferative properties. It induces these effects by binding to and inhibiting normal microtubule turnover leading to cell cycle arrest and apoptosis in different cancer cell lines [1, 2]. While in vitro studies showed 2ME2 to have a high efficacy, in vivo results have been less promising [3, 4]. As a natural metabolite, in vivo processes are geared towards rapid degradation and clearing of 2ME2 from the body [4]. Thus, the use of 2ME2 as a potential treatment for cancer is limited. To circumvent the problems of metabolic processing and the rapid clearing in the liver of 2ME2, new derivatives have been designed and synthesised [5]. Our group designed and synthesised a panel of derivatives in which a sulphamoyl moiety was added to some while for all the methyl group was removed from the two position. Analysis showed that all the sulphamoylated compounds induced mitotic arrest and apoptosis in different cancer cell lines with similar or higher efficacy than 2ME2 [5–7]. However, all but one of the non-sulphamoylated derivatives had no effect on cell survival or cell cycle progression suggesting that the removal of the methoxy group is detrimental for the activity of 2ME2 but this effect is masked by the addition of the sulphamoyl moiety which is a known inhibitor of carbonic anhydrases [8]. Only one non-sulphamoylated derivative, 2-ethyl-estra-1,3,5 (10),15-tetraen-3-ol-17-one, or EE-15-one, affected cancer cell survival with a half maximal effective concentration (EC_50_) in the low micromolar range [8]. EE-15-one is characterized by a ketone group at position C17 which makes EE-15-one an estrone derivative rather than an estradiol derivative. Furthermore, it possesses an alkene group at C15. In this paper we describe the effects and mode of action of this compound. We show that breast cancer cells respond to exposure to EE-15-one by detaching from rigid surfaces through the inhibition of focal adhesions. While cells detach within 2 h, cell death is only slightly increased after 24 h suggesting that EE-15-one does not induce rapid necrosis. Since EE-15-one only inhibits cell adhesion in 2D but not in 3D which is cadherin dependent we suggest that it does not directly interfere with the actin cytoskeleton but rather specifically induces the inactivation and disassembly of focal adhesions. Therefore, EE-15-one is a 2ME2 derivative with a unique mode of action that may have potential as a disruptor of cancer cell metastasis through inhibition of cell–extracellular matrix adhesion.

## Materials and methods

### Reagents

The MCF-7, MDA-MB-231 and BT-20 cell lines were obtained from Cellonex (Johannesburg, South Africa). Most chemical reagents including glutaraldehyde, crystal violet, bovine serum albumin, Tween-20, 4′,6-diamidino-2-phenylindole (DAPI), and Fluoromount aqueous mounting fluid were obtained from Sigma Aldrich (Darmstadt, Germany). NuPAGE LDS sample buffer, MOPS running buffer and 4–12% NuPAGE SDS-PAGE gels were obtained from Invitrogen (Johannesburg, South Africa). The lactate dehydrogenase assay kit II was obtained from Biovision (Johannesburg, South Africa). Total FAK and phospho-S732 FAK antibodies were from Abcam (Pretoria, South Africa). Secondary HRP tagged antibodies were from KPL (Gaithersburg, USA) while the polyvinylidene difluoride (PVDF) membrane was from Amersham (Johannesburg, South Africa). Clarity Western ECL substrate was from Biorad (Johannesburg, South Africa).

### Cell culture

MCF-7 and MDA-MB-231 cells were cultured in GlutaMAX DMEM medium containing 10% fetal calf serum (FCS), while BT-20 cells were cultured in a 1:1 (v/v) DMEM:HamF12 mix containing 10% FCS, and glutamine.

### Cell survival assay

To determine the effect of the compounds on cell viability we used crystal violet staining to determine cell numbers. Exponentially growing cells were seeded in 96-well tissue culture plates (5 × 10^3^ cells/well for MCF-7 and MDA-MB-231 cells, and 1 × 10^4^ cells/well for BT-20 cells) and incubated overnight to allow attachment. The following day cells were exposed to either DMSO (v/v%) or compounds diluted in 200 µl medium. At termination of the experiment cells were fixed with 1% (v/v) glutaraldehyde for 15 min at room temperature (RT). The glutaraldehyde was discarded and cells were stained using 0.1% (w/v) crystal violet at RT for 30 min. The 96-well plate was washed by rinsing the plate in water. Cells were permeabilised and crystal violet solubilised by adding 0.2% Triton X-100 at RT for 30 min. The absorbance of the resulting solution was measured at 570 nm. Six technical repeats were included in each experiment and at least three independent experiments were performed. Graphs represent the average of independent experiments with the error bars representing standard error of the mean. Student’s t-tests were performed to determine statistical significance.

### Cell rounding assay

To quantify the number of cells that round up after exposure to the compounds, cells were seeded in 24 well plates (5 × 10^4^/well) and incubated overnight to attach and spread. Cells were exposed to the compounds after having been photographed and were subsequently photographed at indicated timepoints. At least three images were captured per well and three wells per condition were used in each experiment. Photos were taken at 10× magnification using a Zeiss Primovert microscope and a Zeiss Axiocam ERc5s camera (Zeiss, Oberkochen, Germany). Graphs represent three independent experiments with error bars indicating standard error of the mean.

### Lactate dehydrogenase activity

To quantify the cytotoxicity of our compounds on breast cancer cells the presence of lactate dehydrogenase (LDH) in the medium was analysed using the LDH cytotoxicity assay kit II from Biovision according to the manufacturer’s protocol. In short, MCF-7 and MDA-MB-231 cells were seeded in 96-well cell culture plates (5 × 10^3^/well) and incubated overnight before exposure to 5 µM EE-15-one for the indicated timepoints. Medium from samples (200 μl) was transferred and centrifuged at 2400*g* for 10 min. Afterwards, 10 μl was transferred to a clear 96-well plate. LDH reaction mix (100 μl) was added to the samples and incubated for 90 min at RT. The absorbance was read at 460 nm, with a reference wavelength of 630 nm. Three independent experiments were performed each with three technical repeats. Graphs represent the average of independent experiments with error bars showing standard error of the mean. Statistical significance was determined using the student’s *t*-test.

### Adhesion assay

To determine the effect of compounds on initial cell adhesion and spreading on a rigid surface we performed crystal violet-based adhesion assays. MCF-7 cells were trypsinized and kept in suspension in complete medium. Cells were pre-treated in suspension with either DMSO or 5 µM EE-15-one for 2 h with continuous gentle agitation to prevent cells from aggregating. Cells were subsequently seeded onto plastic culture dishes (96 well, 4 × 10^4^ cell/well) and at indicated time points loose cells were removed while attached cells were fixed in 1% (v/v) glutaraldehyde and processed for crystal violet staining as described previously. Total cell numbers were obtained by collecting 4 × 10^4^ cells by centrifugation and fixing these in glutaraldehyde followed by staining using crystal violet. Three independent experiments were performed each with six technical repeats. Values were calculated as averages with error bars representing standard error of the mean. Statistical significance was calculated using a two-tailed student’s t-test.

### Confocal imaging

Cells were seeded on glass coverslips and incubated overnight to attach and spread. Subsequently, cells were exposed to 5 µM EE-15-one or DMSO for different times before they were fixed in 2% (w/v) paraformaldehyde for 20 min, washed with 1× PBS, permeabilised with 0.2% (v/v) triton X-100 for 5 min, and washed with 1×  PBS. Coverslips were subsequently blocked with 2% (w/v) bovine serum albumin (BSA) in 1× PBS for 1 h, incubated with primary antibodies against FAK for 1 hat RT. Coverslips were washed once more with 1× PBS, and incubated with the secondary antibodies DAPI, and fluorescently conjugated phalloidin for 1 h at RT. After washing with 1× PBS, coverslips were mounted in mounting fluid. Slides were examined using a Zeiss LSM800 Meta confocal microscope furnished with a 63× magnification oil objective.

### Time-lapse imaging

Glass slides (2 cm diameter) were sterilised in 70% ethanol and air-dried in a sterile flow cabinet. The slides were placed into a sterile six well cell culture plate after which cells were seeded on top of the slides at a density of 2.5 × 10^4^ cells per slide. The plates were incubated at 37 °C overnight to allow for attachment. The following day cells were transfected with a paxillin-GFP (Addgene) construct along with a LifeAct-RFP (Addgene) construct. Mirus T2020 transfection reagent was used according to the manufacturer’s protocol. The following day the slides were mounted in the Zeiss life imaging chamber and the microscope incubator set to 37 °C and 5% CO_2_. The Zeiss LSM800 Meta confocal microscope was used to image the slides every 30 s for 2 h at 63× magnification. The images and videos were processed using ImageJ (FIJI) software [9].

### Western blotting

For western blotting 2 × 10^5^ cells were seeded in six well cell culture plates and incubated overnight for attachment. After exposure of EE-15-one for indicated timepoints the medium was removed and collected, and cells were carefully washed with ice-cold 1× PBS. Detached cells were collected by centrifugation and added to adherent cells. Cells were lysed in hot (80 °C) NuPAGE LDS sample buffer supplemented with 2.5% (v/v) β-mercaptoethanol. Cells were scraped and heated at 80 °C for 10 min and centrifuged to remove cell debris. Proteins were separated on 4–12% NuPAGE gels in MOPS running buffer and transferred to polyvinylidene difluoride (PVDF) membranes. After blotting, membranes were blocked in 2% (w/v) BSA and subsequently incubated with anti- β-tubulin, anti-FAK or anti-pFAK primary antibody in BSA for 1 h. The membrane was subsequently washed three times in PBS-T (1× PBS containing 0.5% Tween-20) and incubated with HRP-labelled secondary antibodies for 1 h followed by three more PBS-T washes. Protein expression was analysed using chemiluminescence by incubating membranes in ECL substrate and imaging the membrane using the ChemiDoc MP system (BioRad). Analysis was performed using the ImageLab software from BioRad. Bands were quantified and normalized against β-tubulin protein bands. The graphs represent three independent experiments with error bars as standard deviation. Statistical significance was determined using a two-tailed student’s t-test.

## 3D cell culture and spheroid measurement

BT-20 cells were seeded in DMEM:HamF12 (1:1) media at a density of 2 × 10^4^ per well in a 96-well cell culture plate coated with 1% (w/v) agarose to prevent adherence, as previously described by Friedrich et al. [10]. Spheroids were allowed to form for 4 days after which half the medium was replaced (feeding) before exposure to EE-15-one (5 µM) or DMSO (vehicle control; 0.5% (v/v)) for 72 h. Half of the spheroid medium was replaced on day 7 with fresh medium and every 2 days thereafter. Light micrographs were taken with a Zeiss Axiovert CFL40 fluorescent microscope and Zeiss Axiovert MRm monochrome camera on day 4, 7 and 15, prior to feeding, at 10× magnification.

The area and perimeter of the spheroids were measured using ImageJ software [11, 12] and the volume was determined using three equations [13] including, shape factor (1), spherical volume (2) and shape factor corrected volume (3).

1 $$ \phi = \frac{{{\varvec{\Pi}} \times \sqrt {\frac{{4\varvec{A}}}{{\varvec{\Pi}}}} }}{\varvec{P}} $$

2 $$ \varvec{V} = \frac{{4{\varvec{\Pi}}}}{3}\left( {\frac{\varvec{P}}{{\varvec{\Pi}}}} \right)^{3} $$

3 $$ \varvec{V}^{\varvec{'}} = \phi \varvec{V} $$

Data was normalised to day 4 of each treatment and each point is representative of 60 independent repeats. Sample size was determined using continuous outcome equivalence and superiority power calculations (Sealed Envelope Ltd. 2012, London, UK [14]), where an equivalence limit of 10, 95% power and a 0.025 level of significance were used. Thereafter statistical significance was determined using Student’s t-test where P < 0.05 was deemed significant.

## Results

### EE-15-one exposure affects oestrogen receptor positive and negative breast cancer cell survival

**Fig. 1:**
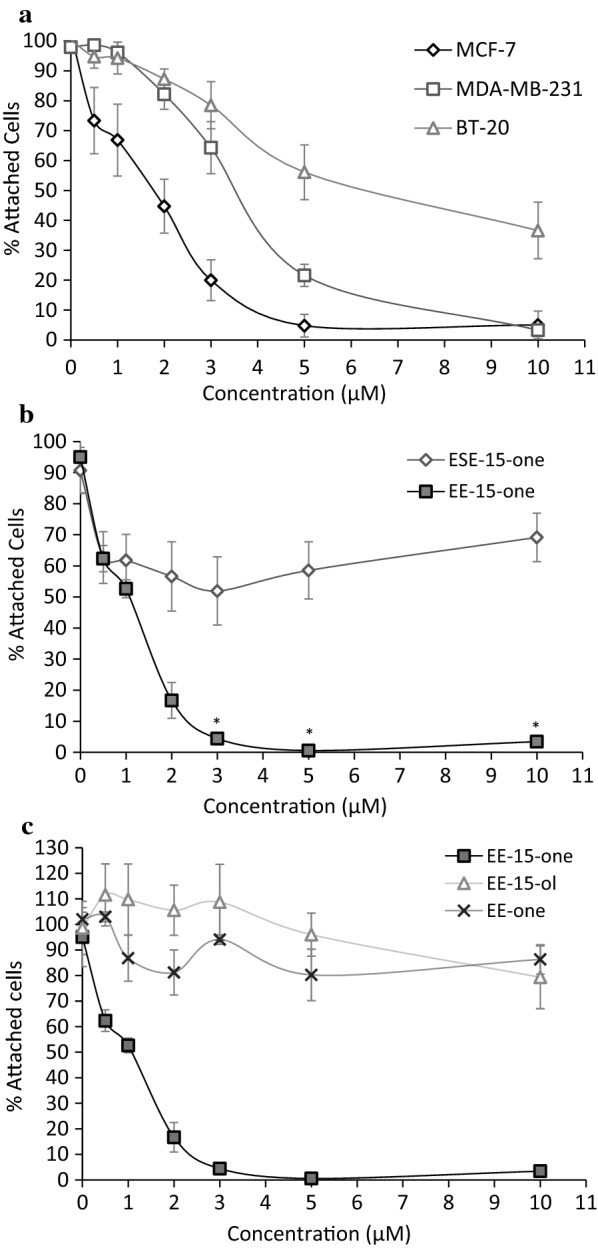
EE-15-one induces rapid loss of cells in different breast cancer cell lines. **a** The effect of EE-15-one on MCF-7, MDA-MB-231 and BT-20 cells was analysed by quantifying cell numbers using crystal violet staining 24 h after exposure to a concentration range of 0.5–10 μM EE-15-one. **b** To determine if EE-15-one had a similar effect on cells as the sulphamoylated ESE-15-one derivative, MCF-7 cells were exposed to a concentration range of either ESE-15-one or EE-15-one and cell number was quantified after 24 h. **c** To identify the essential structural components of EE-15-one responsible for its activity, closely related derivatives lacking either the ketone group at C17 (EE-15-ol) or the alkene group at C15 (EE-one) were added to MCF-7 cells and cell number was quantified using crystal violet staining. All graphs represent three independent experiments with error bars indicating s.e.m. *Represents significant differences as tested by student’s t-test with P < 0.05

To analyse the effect of EE-15-one (Additional file 1: Figure S1) on breast cancer cell line survival, dose response curves were generated by quantifying cell numbers using crystal violet staining and spectrophotometry. ER positive MCF-7, ER negative MDA-MB-231, and ER negative BT-20 cells were exposed to increasing concentrations of EE-15-one for 24 h after which cell numbers were quantified (Fig. 1a). Exposure to micromolar concentrations of EE-15-one led to significant reductions in cell numbers after 24 h. EC_50_ calculations show that MCF-7 cells were most sensitive to EE-15-one with a half maximal effective response (EC_50_) of 1.89 µM whereas MDA-MB-231 cells (EC_50_ = 3.29 µM) and BT-20 cells (EC_50_ = 6.86 µM) were somewhat more resistant.

To compare EE-15-one with other sulphamoylated 2ME2 derivatives that induce cell cycle arrest, dose response curves were performed in MCF-7 cells for EE-15-one and its sulphamoylated homologue, ESE-15-one (Fig. 1b). After 24 h exposure to 5 μM EE-15-one, almost 100% of MCF-7 cells were lost while the maximal cell loss induced by ESE-15-one reached only 43.3%. Therefore, EE-15-one has a significantly greater effect on cell survival than ESE-15-one after 24 h exposure.

To identify the unique structural components of EE-15-one that make it the only non-sulphamoylated 2ME2 derivative to cause significant reduction in cell numbers, closely related derivatives of EE-15-one were analysed for their ability to inhibit breast cancer cell survival. EE-one lacks the alkene group on C15 but contains the ketone group on C17 while EE-15-ol has a hydroxyl group instead of a ketone group on C17 but does contain the alkene group at C15 (Additional file 1: Figure S1). Their effect on MCF-7 cell survival was measured and compared to EE-15-one (Fig. 1c). Neither EE-15-ol nor EE-one had any significant effect on cell numbers after 24 h (vehicle control vs. all concentrations of EE-one or EE-15-ol, P > 0.05) while EE-15-one exposure led to almost complete cell loss at low micromolar concentrations. Thus, both the ketone group at C17 and the alkene group at C15 are needed for the effect of EE-15-one to be present. Our results on MCF-7 cells closely mimic those obtained in other cell lines for these compounds [7, 8].

### EE-15-one rapidly inhibits cell–substrate adhesion

**Fig. 2:**
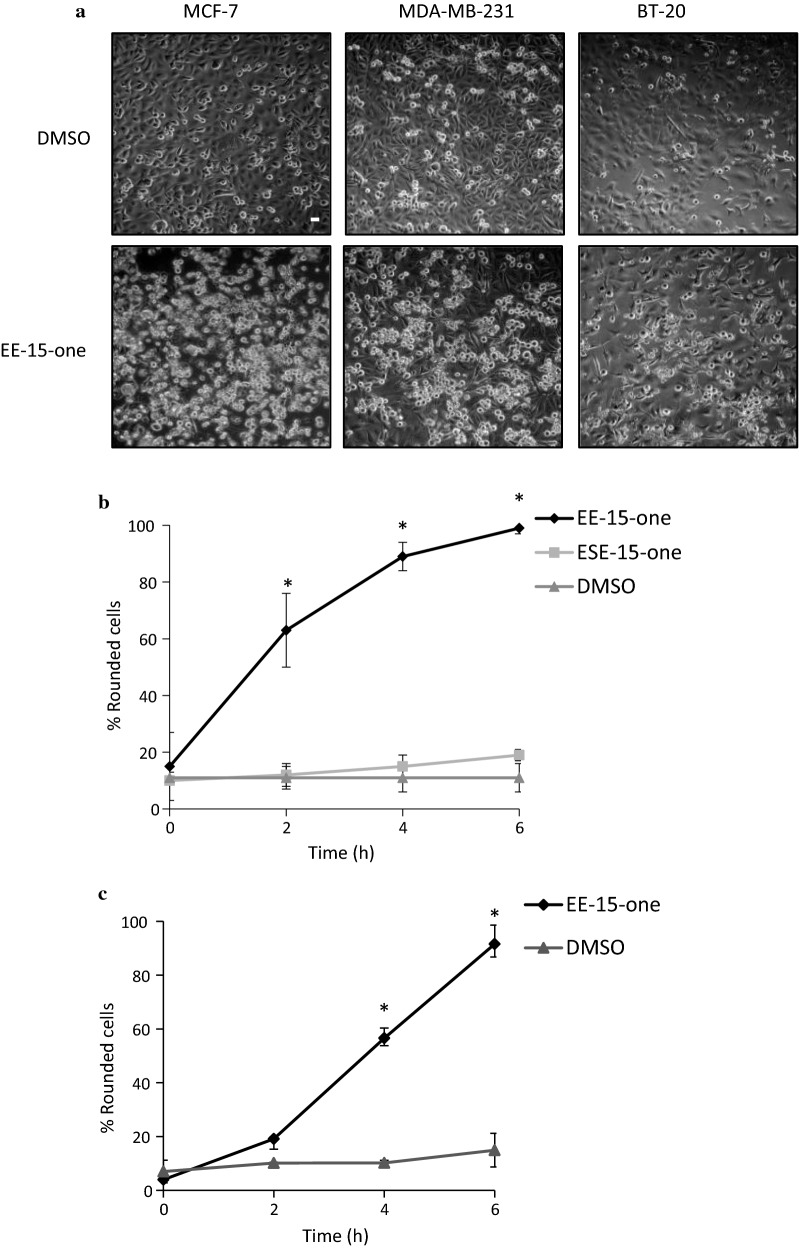
EE-15-one induces rapid cell detachment by inhibiting cell–substrate adhesion. **a** MCF-7, MDA-MB-231, and BT-20 cells exposed to 5 µM EE-15-one for 24 h were imaged by light microscopy using a 10× magnification objective. Scale bar, 10 µm. **b** Cell rounding of MCF-7 cells exposed to 5 µM EE-15-one, 0.5 µM ESE-15-one or DMSO (vehicle control) was quantified by counting the spread and rounded cells in at least 4 fields per condition in three independent experiments. **c** Cell rounding of MDA-MB-231 cells exposed to 5 µM EE-15-one, or DMSO (vehicle control) was quantified by counting the spread and rounded cells in at least 4 fields per condition in three independent experiments. The average of the three independent experiments was plotted while error bars represent the s.e.m. *P < 0.05 in a Student’s *t*-test

To discover the mechanism through which EE-15-one causes the observed rapid loss of cells, we analysed the effect of EE-15-one on cell morphology. Light microscopy images of MCF-7, MDA-MB-231 and BT-20 cells showed that cells became rounded or detached within 24 h after exposure to 5 μM EE-15-one (Fig. 2a). To quantify this observation, MCF-7 and MDA-MB-231 cell morphology were analysed using light microscopy and the number of spread cells and rounded cells were quantified at different times after exposure (Fig. 2b, c). MCF-7 cells exposed to 5 µM EE-15-one rapidly lost their spread morphology with 60% being rounded after 2 h and almost all cells rounded or detached within 4 h. In contrast, neither DMSO nor the sulphamoylated version of EE-15-one, ESE-15-one, induced any significant rounding during this time. In MDA-MB-231 cells 5 µM EE-15-one exposure also resulted rapid cell rounding with almost all cells rounded or detached after 6 h (Fig. 2c). The data suggest that EE-15-one inhibits the ability of cells to remain spread on a rigid surface. Such rapid changes in cell morphology could be the result of the inhibition of cell attachment or of necrosis.

**Fig. 3:**
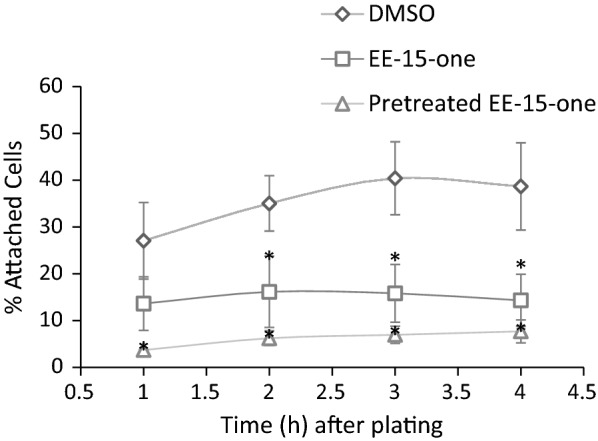
EE-15-one exposure leads to inhibition of cell–substrate adhesion. The effect of EE-15-one on cell–substrate adhesion was quantified by plating suspended MCF-7 cells onto tissue culture treated plates and calculating the number of adhered cells at different times. Suspended cells were either treated with DMSO as control for 2 h, with 5 μM EE-15-one for 2 h or with 5 μM EE-15-one at the time of plating. The average of at least three biologically independent experiments have been averaged with error bars representing s.e.m. *Indicates statistically significant decreases in adhesion (*P *< 0.05, student’s t-test between DMSO and treated)

To determine if EE-15-one impacts on the adhesive ability of cells, initial adhesion and spreading were analysed in MCF-7 cells. To measure the effect of EE-15-one, suspended cells were either treated for 2 h with 5 µM EE-15-one before they were seeded and adhesion was assayed, or 5 µM EE-15-one was added to cells as they were seeded after which they were assayed. Control cells were exposed to DMSO before seeding (Fig. 3). Cells treated for 2 h with EE-15-one lost all adhesive ability with only 5% of seeded cells adhering after 3 h in contrast to 40% of cells adhering when exposed to DMSO. Interestingly, even exposure to EE-15-one at the time of plating led to significant inhibition of adhesion with no more than 15% of plated cells adhering after 3 h. This data suggests that the loss of cells observed after exposure to EE-15-one may be due to an acute inhibition of cell–substrate adhesion.

### EE-15-one does not induce rapid cell necrosis

**Fig. 4:**
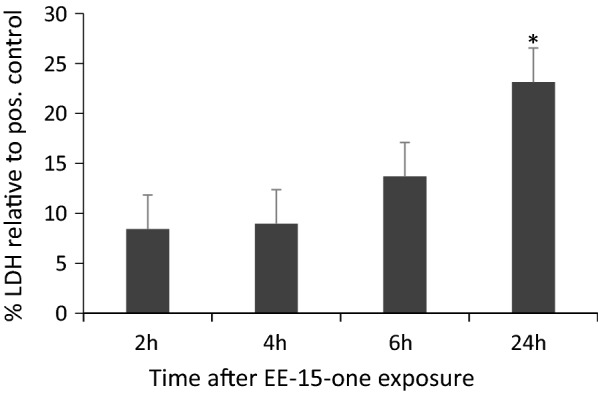
EE-15-one related cell detachment is not due to induced cell death. Medium from MCF-7 cells exposed to 5 μM EE-15-one was collected at the indicated times and analysed for LDH activity. The graph shows the percentage LDH activity as compared to the positive control. The average result of three independent experiments was plotted with error bars representing s.e.m. *Indicates significant differences between negative control medium and medium from EE-15-one treated cells as calculated by student’s t-test at a P < 0.05

Acute loss of adhesion can be due to rapidly induced necrosis. To determine if necrosis was induced by EE-15-one exposure, MCF-7 cellular health was assessed by analysing lactate dehydrogenase (LDH) leakage from cells into the medium which acts as an indicator of a compromised cell membrane signifying necrosis or late stage apoptosis (Fig. 4). Exposure of MCF-7 cells to 5 μM EE-15-one did not lead to significant increases in LDH activity up to 6 h even though all cells were rounded or detached at this time. Only after 24 h was a small but significant increase in LDH activity (20% of positive control) measured. Similarly, LDH activity is only increased significantly after 24 h in MDA-MB-231 cells suggesting that the majority of cells were still alive even though they were rounded or detached (Additional file 2: Figure S2). Therefore, the data suggests that EE-15-one causes rapid cell rounding and detachment most likely through inhibition of cell–substrate adhesion but does not induce rapid and massive necrosis or cell death even after all cells were detached.

### EE-15-one induces the disassembly of focal adhesions with concomitant disruption of the actin cytoskeleton

**Fig. 5:**
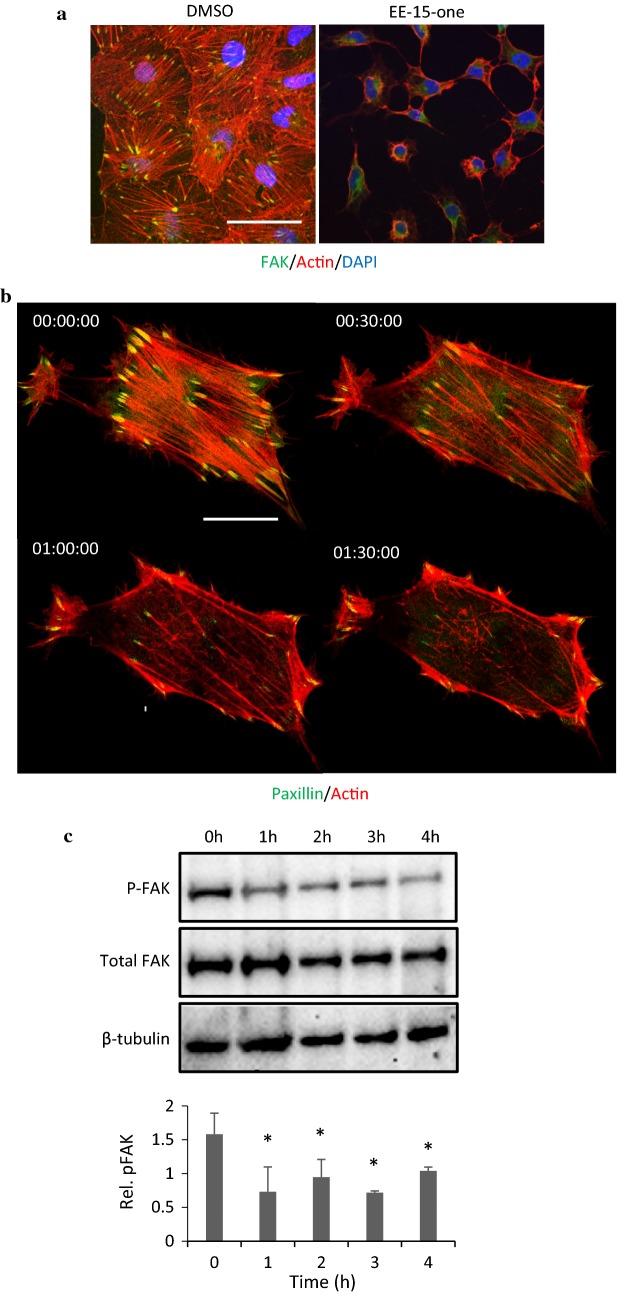
EE-15-one exposure results in the disassembly of the focal adhesions, severance of actin stress fibres connected to focal adhesions, and loss of FAK phosphorylation. **a** MCF-7 cells were treated with 5 µM EE-15-one for 2 h before cells were processed for confocal microscopy. Cells were stained for F-actin (red) and FAK (green) using TexasRed conjugated phalloidin and FAK specific antibodies and FITC labelled secondary antibodies. Cells were imaged using a Zeiss LSM510 confocal microscope with a 63× mag objective, Scale bar, 20 µm. **b** For timelapse imaging, MCF-7 cells were transfected with LifeAct-mRFP (actin, red) and paxillin-GFP (focal adhesions, green). The following day cells were prepared for imaging every 30 s on a Zeiss LSM800 confocal microscope with environmental control. Cells were incubated with 5 µM EE-15-one. Scale bar, 20 µm. **c** MCF-7 cells were treated with 5 µM EE-15-one for the indicated timepoints before being processed for western blot. The top panel shows a representative western blot for phosphorylated FAK, total FAK and β-tubulin as loading control. The graph represents the average ratio of phosphorylated FAK over total FAK with both phosphorylated FAK and total FAK having been normalised to β-tubulin expression. Error bars represent SD and stars denote statistically significant differences between control and treated cells as calculated by student’s t-test with P < 0.05

EE-15-one inhibits cell adhesion and induces cell detachment. Therefore, it is likely that it impacts on focal adhesion function. To determine if EE-15-one does impact on focal adhesions we visualised these along with the actin cytoskeleton. To visualise focal adhesions and the actin cytoskeleton, adherent MCF-7 cells were exposed to DMSO or 5 μM EE-15-one for 2 h before they were fixed and focal adhesions were visualised with an anti-FAK antibody (green) while the actin cytoskeleton was visualised using fluorescently labelled phalloidin (red) (Fig. 5a). DMSO-treated cells possessed multiple focal adhesions throughout the basal membrane which were connected to well-defined, thick actin stress fibres. However, EE-15-one treated cells were poorly spread with long protrusions. No clear FAK positive structures were visible and instead a general cytoplasmic staining was observed. The actin cytoskeleton was almost completely lost with staining only visible close to the cell membrane and no observable stress fibres. Since the effect of EE-15-one was rapid, real time imaging was used to investigate the dynamics of the actin cytoskeleton and the focal adhesions after EE-15-one exposure (Fig. 5b, Additional file 3: Video S3). MCF-7 cells were transfected with paxillin-GFP and actin-mRFP constructs and were filmed using confocal microscopy. Still images of co-transfected MCF-7 cells show that within 1 h after exposure to 5 μM EE-15-one, actin fibres were becoming severed and stress fibres started thinning. After 1.5 h most stress fibres were lost with only cortical actin fibres remaining. At the same time, focal adhesions rapidly reduced in size so that at 1.5 h many focal adhesions had completely disassembled. Together, these data suggest that EE-15-one induces the disassembly of focal adhesions and the loss of tension through actin stress fibres. To analyse if EE-15-one exposure leads to the inhibition of focal adhesion signalling, FAK Y397 phosphorylation, which acts as a marker for FAK activation, was quantified by western blot (Fig. 5c). Western blot analysis using antibodies targeting phosphorylated FAK, total FAK and β-tubulin as loading control were used to assess the phosphorylation status of FAK in control cells and cells exposed to 5 µM EE-15-one for 1–4 h (Fig. 5c). The results show that EE-15-one significantly reduced FAK phosphorylation even after 1 h exposure to EE-15-one. Phosphorylation remained diminished throughout the time course while total FAK levels remained constant. Together, these data suggest that EE-15-one impacts on cell adhesion by abrogating focal adhesion function along with a loss of tension via the actin cytoskeleton leading to cell rounding and detachment.

### EE-15-one does not inhibit cadherin dependent cell–cell adhesion

**Fig. 6:**
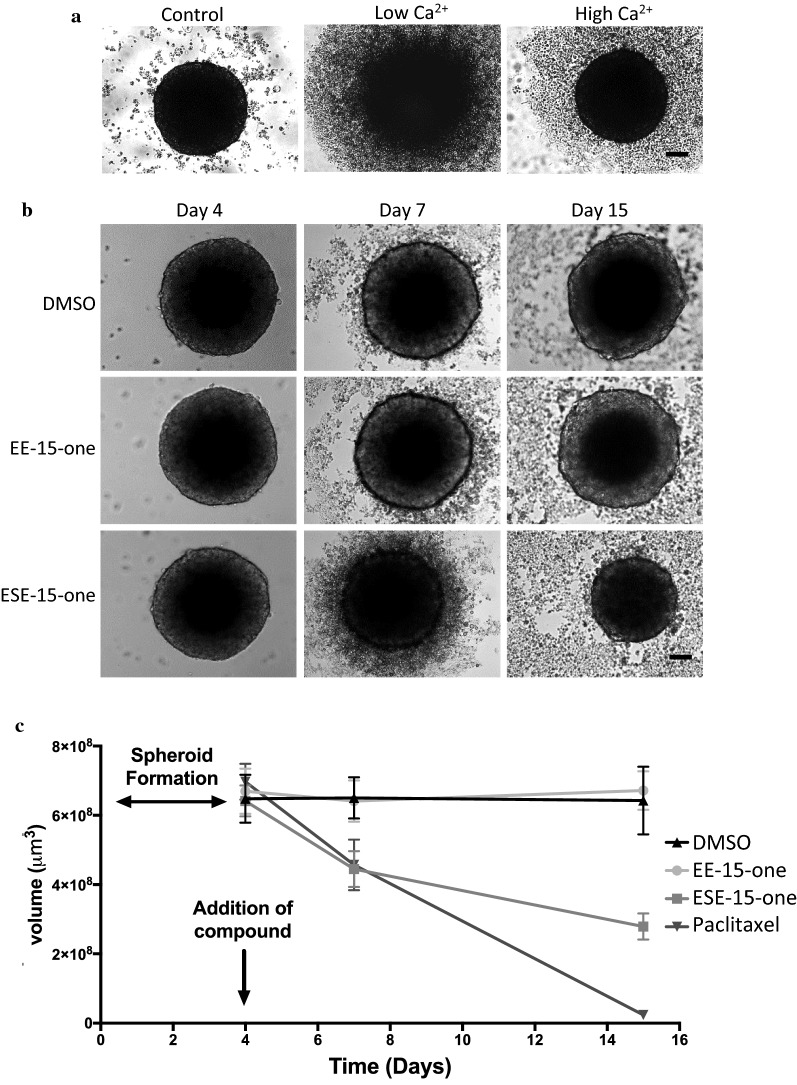
EE-15-one does not affect cadherin dependent cell–cell adhesion. **a** To show that cell spheroids aggregate through Ca^2+^-dependent cell–cell adhesion, BT-20 cells (2 × 10^4^) were seeded in 1% agarose coated wells and allowed to form for 4 days as described in the materials and methods. Spheroids were then exposed to water (control), 2 mM EGTA (Low Ca^2+^), or 2 mM EGTA and 4 mM CaCl_2_ (high Ca^2+^). Light microscopy images were taken after 24 h. **b** BT-20 spheroids were exposed to EE-15-one (5 µM), ESE-15-one (1 μM), 500 nM paclitaxel as positive control or DMSO (vehicle control). **c** The area and perimeter of the spheroid were measured and used to determine volume. Day 4 was set as staring volume and at each point the average of 60 independent spheroids volumes are represented. Error bars indicate standard deviation. Light microscopy images were taken at day 4, 7 and 15 at 10× magnification. Scale bar = 100 µm

EE-15-one exposure leads to rapid cell detachment from the tissue culture surface which is dependent on the formation of functional focal adhesions. Whether EE-15-one directly impacts on focal adhesions or on the actin cytoskeleton is unclear from the previous experiments. Therefore, we tested the ability of EE-15-one to interfere with adhesion in a setting in which integrin dependent adhesion was absent. Epithelial cell adhesion in three dimensional settings such as cell spheroids does not depend on cell–substrate adhesions mediated by integrins but rather on Ca^2+^-dependent cadherin adhesions or adherens junctions [15]. Importantly, both integrin based focal adhesions and cadherin based adherens junctions link to the actin cytoskeleton. Therefore, we hypothesised that if EE-15-one is an actin disruptor, cancer cell spheroids would be affected after exposure to EE-15-one. Due to a lack of effective spheroid formation using MCF-7 and MDA-MB-231 cells, BT-20 cell spheroids were generated by growing cells in non-adhesive cell culture plates. BT-20 cells formed compact, well-defined spheroids over 4 days (Fig. 6a, control). To show that these spheroids do indeed depend on Ca^2+^ dependent adhesions, they were cultured for 24 h in the presence of the Ca^2+^ chelator EGTA (Fig. 6a, low Ca^2+^). The well-defined spheroid border was lost, and numerous single cells were spread around the remnants of the spheroid suggesting that chelating Ca^2+^ led to reduced adhesion within the spheroid. However, when spheroids were cultured with EGTA and an excess of Ca^2+^, the border of the spheroid remained well-defined and the spheroid was intact (Fig. 6a, high Ca^2+^). Therefore, BT-20 spheroids are assembled by Ca^2+^ dependent cell–cell adhesion. To test if EE-15-one inhibits cell–substrate adhesion or rather impacts on the actin cytoskeleton, BT-20 spheroids were exposed to DMSO as control, 5 μM EE-15-one, 1 μM ESE-15-one as a control for compound exposure and 500 nM paclitaxel as positive control (Fig. 6b). Images of spheroids exposed to DMSO and EE-15-one showed well-defined spheroids with a smooth spheroid border that remained similar in size over the measured timeframe. In contrast, ESE-15-one exposed spheroids lost the smooth spheroid edge and diminished in size. Spheroid volumes were quantified at the time of exposure, and 3 and 11 days after exposure. Interestingly, while BT-20 cells grown in monolayer detached significantly within 24 h after exposure to 5 μM EE-15-one (Figs. 1, 2a), exposure of spheroids to 5 μM EE-15-one had no effect on spheroid volume at any time. In contrast, the sulphamoylated derivative ESE-15-one caused a significant reduction in spheroid volume similarly to the paclitaxel positive control. Therefore, the spheroid volume data suggests that EE-15-one is affecting cells attached via cell–substrate adhesion while it does not affect cells adhering via cell–cell adhesion.

## Discussion

The oestrogen metabolite, 2ME2, inhibits cancer cell growth by affecting microtubule dynamics leading to cells arresting at the G2/M border of the cell cycle culminating in apoptosis [1, 2, 16, 17]. However, in vivo studies indicated that 2ME2 was rapidly metabolised and cleared from the blood by the liver [3, 4]. Previously, a panel of 2ME2 derivatives were designed in our group in which the methoxy group on position 2 was replaced with an ethyl group and half of the compounds were also modified with a sulphamoyl moiety [5]. Interestingly, all the sulphamoylated compounds showed antiproliferative activity against different cancer cell lines [8]. However, almost all the non-sulphamoylated compounds had no activity at all suggesting that replacing the 2-methoxy group with an ethyl group abrogated the compounds’ antiproliferative effect. One non-sulphamoylated compound, called EE-15-one, did reduce cell numbers in different cancer cell lines with an EC_50_ in the low micromolar range [8]. Therefore, we suggest that this is the only true 2ME2 derivative with anti-proliferative capability. In this study we set out to understand the mode of action of EE-15-one to characterise it and compare it to the original compound 2ME2.

Our data show that exposure to EE-15-one in three different breast cancer cell lines resulted in the loss of cells to a maximal effect of 100% in MCF-7 and MDA-MB-231 cell lines after 24 h. This effect on cell number is in contrast to the published effects of 2ME2 and the sulphamoylated derivatives which only induce a partial loss of cells even after 48 h [7]. Sulphamoylated compounds including ESE-15-one decrease cell numbers by a maximum of 70% after 48 h which suggests that EE-15-one induces a different effect in cells than 2ME2 or sulphamoylated derivatives. Comparison between EE-15-one and the sulphamoylated version, ESE-15-one, showed that while EE-15-one induced a rapid and almost complete loss of cells, ESE-15-one only reduced cell numbers partially. This suggests that addition of the sulphamoyl moiety repurposes the molecule and changes its mode of action. Therefore, EE-15-one is a unique 2ME2 derivative that induces cell loss through a mechanism which is different to that induced by 2ME2 or the sulphamoylated derivatives.

Our data shows that exposure to EE-15-one causes rapid cell rounding in all three tested cell lines. In fact, quantifying cell rounding in MCF-7 and MDA-MB-231 cells shows that EE-15-one exposure induces cell rounding within 2 h while exposure to ESE-15-one did not induce any cell rounding within the same timeframe. Thus, we suggest that EE-15-one is a non-sulphamoylated 2ME2 derivative that causes cell loss by inducing cell detachment.

Cell rounding and attachment is mediated by the regulation of cell–substrate adhesion via the focal contacts. Our data show that exposure before or at the time of seeding blocks cell adhesion almost completely. This data suggest that cell–substrate adhesion normally mediated by cell membrane receptors such as integrins is lost. Microscopy and biochemical analysis further substantiate our assessment that EE-15-one interferes in cell–substrate adhesion. Firstly, visualisation of focal adhesions and the actin cytoskeleton by confocal microscopy shows that focal adhesions rapidly disassemble after EE-15-one exposure while the actin stress fibres become severed and disorganised. This will result in a loss of tension and adhesive ability needed for proper adhesion. Secondly, biochemical analysis of focal adhesion signalling showed that signalling is rapidly diminished and remains so over many hours. Cadherins are homotypic cell adhesion molecules responsible for cell–cell adhesion and, like integrins, adhere to extracellular ligands while intracellularly they link to the actin cytoskeleton. To determine if EE-15-one impacts on the actin cytoskeleton rather than on the focal adhesions, we tested the effect of EE-15-one on cells grown in spheroids. BT-20 spheroids depend on Ca^2+^-mediated cadherin adhesion to form while integrin adhesion in this setting has not been reported [15]. Our data show that in this setting EE-15-one no longer induces the loss of adhesion. This suggests that EE-15-one does not affect the actin cytoskeleton directly, but rather specifically inhibits cell–substrate adhesion through the induction of focal adhesion disassembly. Lastly, we show that the effects of EE-15-one on adhesion is not due to a necrotic effect of the compound. LDH activity in the medium shows only a small increase 24 h after exposure at which time all cells have been detached for more than 12 h. Therefore, cell detachment precedes this small increase in necrosis. It is worth noting that although rapid autophagy [18] and apoptosis [19, 20] have been reported after cell detachment, no membrane blebbing, hyper condensation of chromatin or nucleosome release could be noted post EE-15-one exposure. However, further investigation into apoptotic cascades such as the various caspases, Bid, Bax and Bcl-2 activation could be performed. Increased autophagy has also been linked to anoikis resistance in cancer cells [21]. Future studies will focus on this phenomenon related to the induced loss of cell adhesion by EE-15-one.

## Conclusions

We have identified a unique 2ME2 derivative that elicits a potent and rapid loss of cancer cells. We show that unlike 2ME2 which acts through the inhibition of microtubule dynamics and a concomitant arrest of the cell cycle, EE-15-one induces the rapid disassembly of focal adhesions leading to cell–substrate detachment. This compound could be a potent inhibitor of breast cancer cell metastasis as it inhibits cell–substrate adhesion which is essential for local invasion and dissemination.

## Additional files

**Additional file 1: Figure S1.** Chemical structure of EE-15-one. The chemical structure of 2-Ethyl-estra-1,3,5(10),15-tetraen-3-ol-17-one (EE-15-one) showing the ketone group at position C17 which makes EE-15-one an estrone derivative rather than an estradiol derivative along with an alkene group at C15. Additionally, the chemical structures of ESE-15-one, EE-one, EE-15-ol are depicted.

**Additional file 2: Figure S2.** EE-15-one related cell detachment in MDA-MB-231 cells is not due to induced cell death. Medium from MDA-MB-231 cells exposed to 5 μM EE-15-one was collected at the indicated times and analysed for LDH activity. The graph shows the percentage LDH activity as compared to the positive control. The average result of three independent experiments was plotted with error bars representing s.e.m. *Indicates significant differences between negative control medium and medium from EE-15-one treated cells as calculated by student’s t-test at a P < 0.05.

**Additional file 3: Video S1.** EE-15-one exposure results in the disassembly of the focal adhesions, severance of actin stress fibres connected to focal adhesions. For timelapse imaging, MCF-7 cells were transfected with LifeAct-mRFP (actin, red) and paxillin-GFP (focal adhesions, green). The following day cells were prepared for imaging every 30 s on a Zeiss LSM800 confocal microscope using a 63× magnification objective with environmental control. Cells were incubated with 5 µM EE-15-one. Scale bar, 20 µm.

## Abbreviations

2ME2: 2-methoxyestradiol
BSA: bovine serum albumin
DAPI: 4′,6-diamidino-2-phenylindole
DMSO: dimethylsulfoxide
EC_50_: half maximal effective concentration
EE-15-one: 2-ethyl-estra-1,3,5 (10),15-tetraen-3-ol-17-one
ER: estrogen receptor
FAK: focal adhesion kinase
FCS: fetal calf serum
h: hour
LDH: lactate dehydrogenase
PVDF: polyvinylidene difluoride
RT: room temperature
SDS-PAGE: sodium dodecyl sulfate polyacrylamide gel electrophoresis
