# Supplementary figures and images for: A novel non-sulphamoylated 2-methoxyestradiol derivative causes detachment of breast cancer cells by rapid disassembly of focal adhesions
Source: Cancer Cell Int. 2018 Nov 19;18:188. doi: 10.1186/s12935-018-0688-7 (PMC6245879; doi:10.1186/s12935-018-0688-7)

## Slide 1
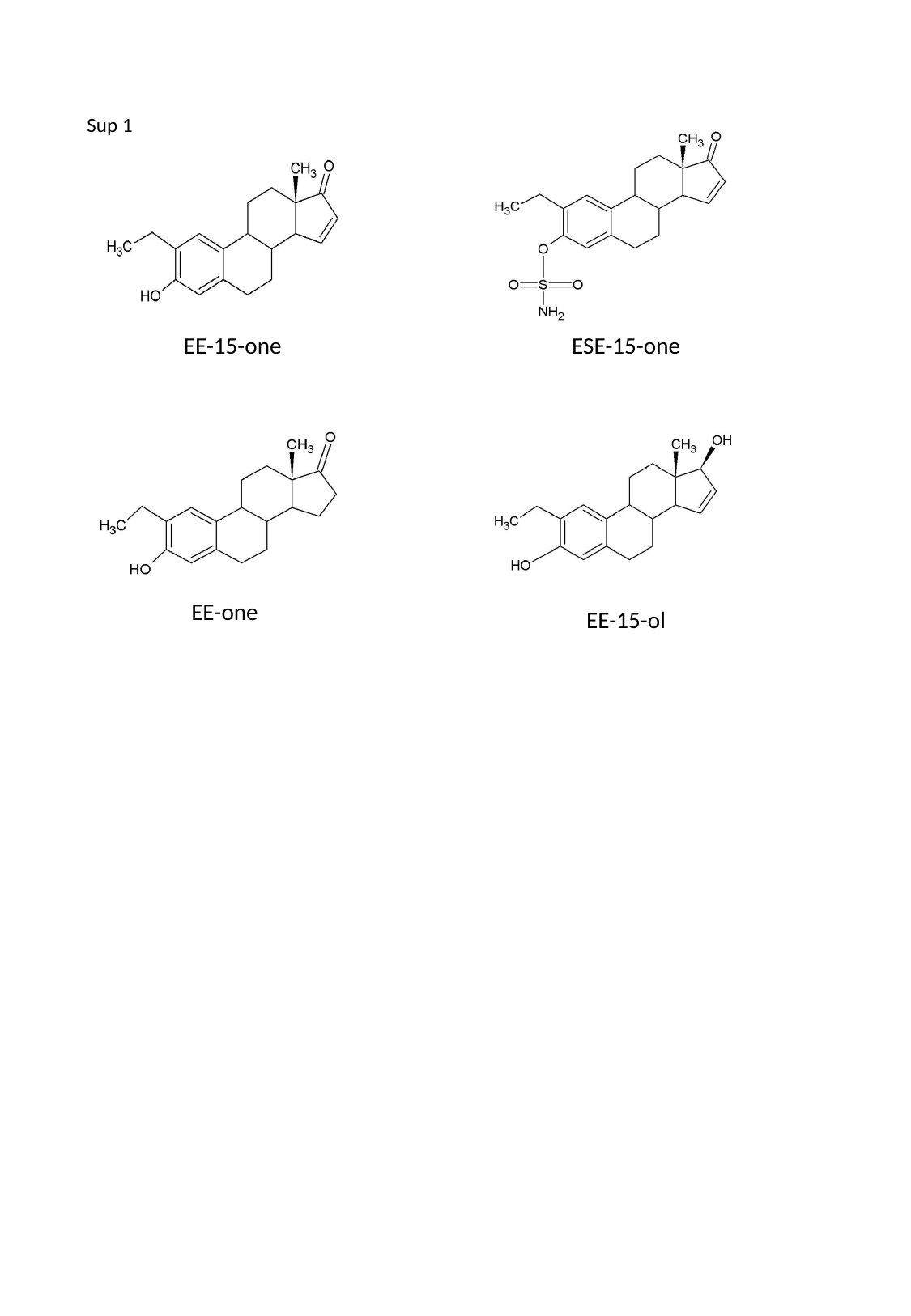

Sup 1
EE-15-one
ESE-15-one
EE-one
EE-15-ol

Supplement: Supplementary file 1 — Additional file 1: Figure S1. Chemical structure of EE-15-one. The chemical structure of 2-Ethyl-estra-1,3,5(10),15-tetraen-3-ol-17-one (EE-15-one) showing the ketone group at position C17 which makes EE-15-one an estrone derivative rather than an estradiol derivative along with an alkene group at C15. Additionally, the chemical structures of ESE-15-one, EE-one, EE-15-ol are depicted. [file 12935_2018_688_MOESM1_ESM.pptx]

## Slide 1
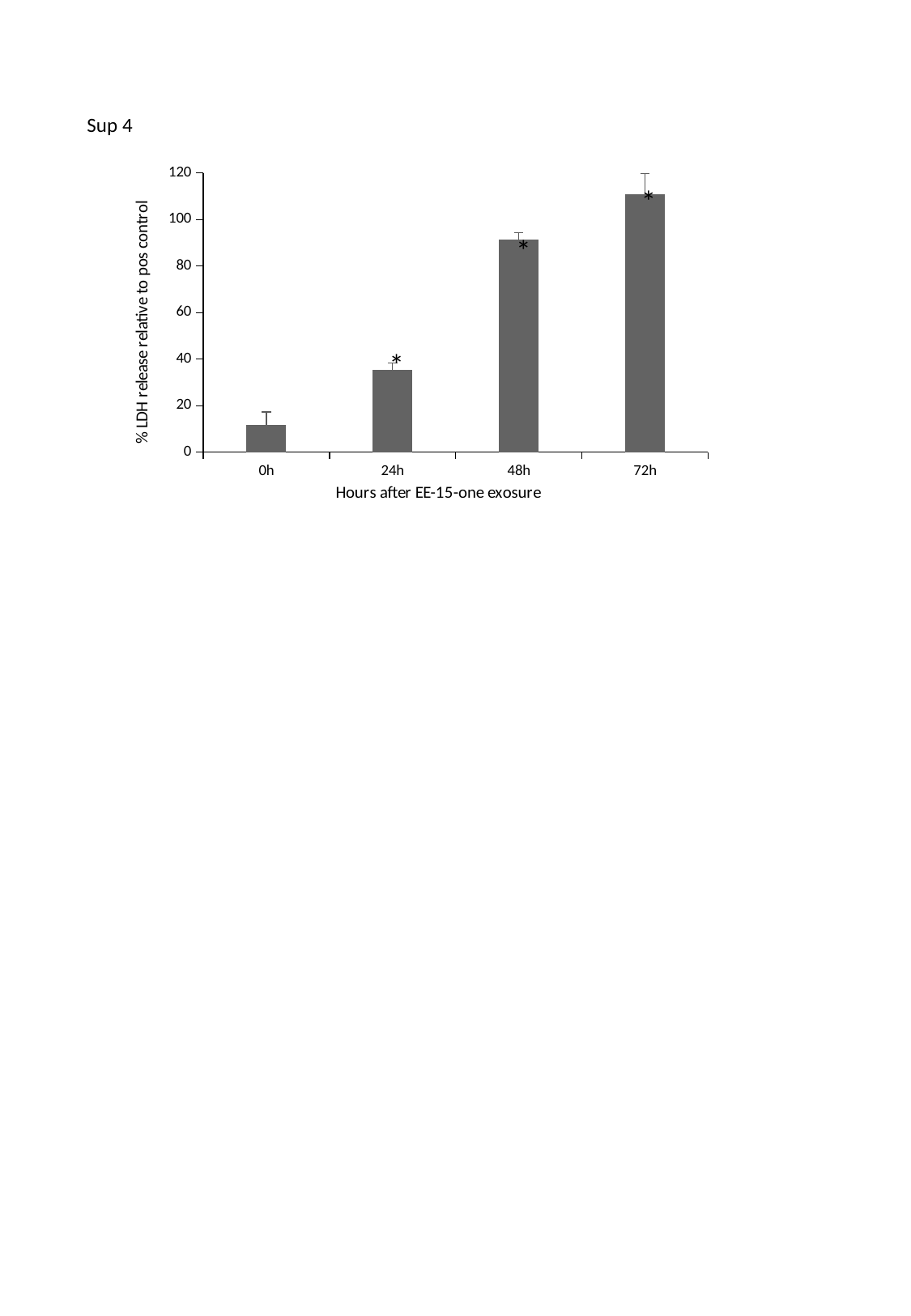

Sup 4
### Chart
| Category | |
|---|---|
| 0h | 11.506550978290456 |
| 24h | 35.266872927697776 |
| 48h | 91.41933270994349 |
| 72h | 110.8211870668812 |*
*
*

Supplement: Supplementary file 2 — Additional file 2: Figure S2. EE-15-one related cell detachment in MDA-MB-231 cells is not due to induced cell death. Medium from MDA-MB-231 cells exposed to 5 μM EE-15-one was collected at the indicated times and analysed for LDH activity. The graph shows the percentage LDH activity as compared to the positive control. The average result of three independent experiments was plotted with error bars representing s.e.m. *Indicates significant differences between negative control medium and medium from EE-15-one treated cells as calculated by student’s t-test at a P < 0.05. [file 12935_2018_688_MOESM2_ESM.pptx]
